# Microplastics in Human Thoracic Tissues: Increased Burden in Patients with Lung Malignancy

**DOI:** 10.3390/jcm15176503

**Published:** 2026-08-22

**Authors:** Ilias E. Dimeas, George E. Zakynthinos, Christos Salmas, Ioannis Diamantis, Maria Giannaki, Charalampos Varsamas, Achilleas Sarris, Angeliki Miziou, Paraskevi Kirgou, Sotirios I. Sinis, Kathryn Duvall, Stavros P. Anastopoulos, Cormac McCarthy, Patrick D. Mitchell, Michael P. Keane, Maria Perraki, Evangelos Oikonomou, Ioannis D. Papanikolaou, Zoe Daniil

**Affiliations:** 1 Department of Respiratory Medicine, University Hospital of Larissa, Faculty of Medicine, School of Health Sciences, University of Thessaly, 41110 Larissa, Greece; 2 School of Medicine, University College Dublin, D04V1W8 Dublin, Ireland; 3 3rd Department of Cardiology, “Sotiria” Chest Diseases Hospital, Medical School, National and Kapodistrian University of Athens, 10679 Athens, Greece; 4 Laboratory of Mineralogy-Geology, Department of Natural Resources and Agricultural Engineering, Agricultural University of Athens, 11855 Athens, Greece; 5 Department of Cardiothoracic Surgery, University Hospital of Larissa, 41110 Larissa, Greece; 6 Department of Geology and Geoenvironment, National and Kapodistrian University of Athens, 10679 Athens, Greece; 7 Department of Respiratory Medicine, St. Vincent’s University Hospital, D04T6F4 Dublin, Ireland; 8 Respiratory Department, Tallaght University Hospital, D24NR0A Dublin, Ireland; 9 School of Medicine, Trinity College Dublin, D02PN40 Dublin, Ireland; 10 Division of Geo-Sciences, School of Mining and Metallurgical Engineering, National Technical University of Athens, 15780 Athens, Greece; 11 3rd Cardiology Department, “Sotiria” General Hospital of Chest Diseases of Athens, Medical School, National and Kapodistrian University of Athens, 10679 Athens, Greece

**Keywords:** microplastics, lung biopsy, lung neoplasms, medical thoracoscopy, environmental exposure

## Abstract

**Background:** Human respiratory exposure to microplastics (MPs) has recently been demonstrated, but their distribution within thoracic tissues and association with lung malignancy remain poorly characterised. We investigated the presence, burden, morphology, and spectroscopic characteristics of MPs in thoracic tissue specimens and their relationship with lung malignancy. **Methods:** In this prospective observational study, 50 adults undergoing diagnostic thoracic surgical procedures for suspected lung malignancy were enrolled. Lung, pleural, and mediastinal lymph node specimens underwent contamination-controlled processing, microscopy, and representative particle analysis by confocal micro-Raman spectroscopy. Associations between MPs and clinicopathological variables were analysed. **Results:** Sixty-two morphologically identified microplastic particles were detected in 50 specimens, with MPs detected in 32 patients (64%). Fragments accounted for 97% of particles, with a median burden of one particle per specimen (IQR, 2). MP detection was significantly more frequent in patients with primary lung malignancy than in patients without primary lung malignancy, including those with metastatic malignancy and benign disease (81.8% vs. 50.0%, *p* = 0.020), who also exhibited a higher tissue MP burden (*p* = 0.024). Primary lung malignancy remained independently associated with MP detection after adjustment for age and smoking exposure (OR 4.09, 95% CI 1.06–15.79; *p* = 0.041). Particle morphology varied by biopsy site, with significantly smaller particles identified in mediastinal lymph nodes (*p* = 0.006). Synthetic anthropogenic spectral signatures were identified in 71% of particles. **Conclusions:** MPs are frequently present in human thoracic tissues and are associated with primary lung malignancy. Compartment-specific particle characteristics suggest distinct patterns of respiratory MP deposition and retention, supporting multicentre mechanistic studies.

## 1. Introduction

Microplastics (MPs), defined as plastic particles smaller than 5 mm, have emerged as pervasive environmental contaminants following the exponential growth of global plastic production and disposal. Their widespread presence in indoor and outdoor air has established inhalation as an important route of human exposure, with computational and experimental studies demonstrating deposition throughout the respiratory tract according to particle size, morphology, density, and aerodynamic characteristics [1,2]. Atmospheric transport studies have further demonstrated that airborne MPs are widely dispersed and can be deposited even in remote environments, underscoring the ubiquity of inhalational exposure [3]. In addition to their intrinsic physicochemical properties, MPs may act as carriers of adsorbed contaminants, including heavy metals and persistent organic pollutants, potentially modifying their biological behaviour after deposition within the lung [2,4]. Increasing attention has therefore focused on their potential impact on respiratory health.

Unlike many conventional airborne pollutants, MPs constitute a highly heterogeneous group of particles that differ substantially in size, shape, surface chemistry, and polymer composition, all of which may influence their aerodynamic behaviour and biological interactions after inhalation [1,2,4]. Fragmented particles and fibres exhibit distinct deposition patterns within the respiratory tract, while surface ageing and adsorption of environmental contaminants may further modify their toxicity [2,4]. Consequently, the respiratory effects of MPs are unlikely to be explained solely by particle burden, highlighting the importance of characterising particle morphology and other physicochemical features alongside quantitative measurements.

Direct evidence of respiratory exposure has recently emerged through the detection of MPs in human biological samples. MPs have been identified in surgically resected lung tissue [5,6], bronchoalveolar lavage (BAL) fluid [7,8,9,10], and multiple human tumour tissues, including primary lung cancer [11,12,13,14], indicating that inhaled particles reach the distal respiratory tract and may accumulate according to disease state and tissue characteristics. Nevertheless, most available studies have examined a single respiratory compartment, providing only a partial representation of pulmonary MP deposition. In contrast, surgically obtained thoracic tissues offer the opportunity to evaluate retained particles across distinct anatomical compartments, including the lung parenchyma, pleura, and mediastinal lymph nodes, thereby providing complementary information regarding particle deposition, retention, and potential translocation within the thorax. This compartmental approach remains largely unexplored in human studies.

Thoracic tissues obtained during diagnostic surgical procedures provide a unique opportunity to investigate MPs within anatomically distinct compartments that differ in their physiological function and mechanisms of particle handling. Whereas lung parenchyma represents the primary site of inhaled particle deposition, the pleura and mediastinal lymph nodes may reflect secondary pathways of particle retention, clearance, and lymphatic translocation [15,16]. Evaluating these tissues within the same clinical cohort may therefore provide insights into the compartment-specific distribution of MPs that cannot be obtained from studies examining a single specimen type. Such information may improve understanding of how inhaled MPs interact with the human respiratory system following long-term environmental exposure.

Although MPs have been detected in human respiratory tissues, their biological significance remains uncertain. Experimental studies demonstrate oxidative stress, epithelial injury, chronic inflammation, macrophage dysfunction, immune dysregulation, and activation of carcinogenesis-related pathways, but current clinical evidence remains observational and does not establish causality [14,17,18,19,20,21,22,23]. Experimental and computational models further indicate that particle size is a major determinant of pulmonary deposition, retention, and translocation, with smaller particles demonstrating greater potential to migrate across biological barriers and enter lymphatic pathways [1,15,16]. Together, these observations provide biological plausibility for tissue accumulation while emphasising the need for well-characterised human studies.

Most human studies have included small cohorts, heterogeneous analytical methodologies, and a single specimen type, limiting comparisons and leaving the distribution of MPs across thoracic tissues and their clinicopathological correlates incompletely characterised [5,6,7,8,9,10,11,12,13,14]. Lung malignancy provides a clinically relevant setting in which to investigate respiratory MP accumulation because malignant transformation alters tissue architecture, vascular permeability, inflammatory signalling, extracellular matrix remodelling, and immune-cell composition, potentially influencing particle deposition, retention, and clearance independently of environmental exposure [12,17,21]. Furthermore, comparing malignant and non-malignant thoracic tissues obtained through the same diagnostic pathway enables assessment of whether differences in MP burden reflect disease-associated tissue characteristics rather than exposure alone.

Therefore, a prospective observational study was conducted in adults undergoing diagnostic thoracic surgical procedures for suspected lung malignancy. Using rigorous contamination-control procedures together with representative Raman spectroscopic confirmation, the presence, burden, morphology, and spectroscopic characteristics of MPs were characterised in lung, pleural, and mediastinal lymph node biopsy specimens. The primary objective was to determine whether tissue MP burden and detection differed between patients with primary lung malignancy and patients without primary lung malignancy. Secondary objectives were to investigate compartment-specific differences in MP characteristics across thoracic tissues and to explore associations between MP detection and clinicopathological features.

## 2. Materials and Methods

### 2.1. Study Design and Participants

This prospective observational study enrolled 50 consecutive adults undergoing diagnostic thoracic surgical procedures at the University Hospital of Larissa, Greece, between May and August 2025. Written informed consent was obtained from all participants. Ethical approval was granted by the Human Research Ethics Committee of the University Hospital of Larissa (approval no. 22/6th; 29 April 2025), and the study was conducted in accordance with the Declaration of Helsinki. The study is reported according to the Strengthening the Reporting of Observational Studies in Epidemiology (STROBE) guidelines.

Eligible participants underwent diagnostic thoracic tissue sampling for suspected lung malignancy, including surgical lung biopsy, mediastinal or hilar lymph node biopsy, or medical thoracoscopy with pleural biopsy. Consecutive patient recruitment was used to minimise selection bias, and all eligible participants during the study period were invited to participate. Histopathological diagnoses were established according to routine clinical practice by experienced thoracic pathologists who were blinded to the microplastic analyses. Patients with a history of self-reported occupational plastic exposure, previous chemotherapy or radiotherapy, or another active malignancy were excluded. Occupational plastic exposure was defined as work involving the manufacture, processing, recycling, or industrial handling of plastics, polymers, or synthetic fibres likely to generate airborne exposure above usual community levels and was assessed from the occupational history obtained at enrolment. No quantitative occupational exposure assessment was performed. To minimise variability in environmental exposure, only participants who self-reported residence in the Thessaly region for more than 50% of their lifetime and considered it their permanent residence were included.

### 2.2. Sample Collection, Contamination Control and Sample Processing

Fresh thoracic tissue specimens were collected immediately after excision and confirmed by the attending pathologist to be diagnostically adequate before analysis. The specimen allocated for microplastic analysis was obtained from the same diagnostic biopsy or surgical sampling procedure as the tissue submitted for routine histopathological evaluation and was selected by the attending pathologist to be representative of the sampled lesion or anatomical site. Because tissue allocated for microplastic analysis underwent contamination-controlled processing rather than histopathological examination, the microscopic composition of the analysed fragment was not independently verified. Consequently, MP findings were interpreted according to each patient’s final clinicopathological diagnosis rather than the histology of the specific tissue fragment analysed. Demographic data, histological diagnosis, and biopsy type were recorded for each participant. Peripheral blood samples were analysed for routine haematological and biochemical parameters as part of standard clinical care. Variables recorded for analysis included haemoglobin concentration, white blood cell count and differential (neutrophils, lymphocytes, monocytes, eosinophils and basophils), platelet count, mean corpuscular volume (MCV), C-reactive protein, serum albumin, lactate dehydrogenase and the neutrophil-to-lymphocyte ratio. These variables were selected because they reflect systemic inflammation, immune status, tissue injury, and general physiological condition, and were explored for potential associations with tissue microplastic detection and burden.

Rigorous contamination-control measures were implemented throughout specimen collection, processing, and analysis to minimise environmental contamination. Samples were collected in pre-cleaned glass containers, all reagents were pre-filtered before use, and all procedures were performed within a laminar-flow cabinet using dedicated glass laboratory equipment. Plastic consumables were avoided throughout sample processing whenever suitable alternatives were available, and laboratory personnel wore cotton laboratory coats and powder-free nitrile gloves throughout sample handling. Individual specimens were processed separately to minimise cross-contamination, and all work surfaces and equipment were thoroughly cleaned before each analysis.

Organic material was digested with 10% potassium hydroxide at a sample-to-reagent ratio of 1:5 and incubated at 55 °C for 48 h. Digested samples were vacuum-filtered through cellulose nitrate membrane filters (0.47 μm pore size), which were dried in covered glass Petri dishes before systematic examination of the entire membrane surface by stereomicroscopy. Suspected MPs were subsequently evaluated by transmitted-light microscopy. Maximum particle diameter (Dmax) and minimum Feret diameter (DFeretmin) were measured, and particle colour was recorded according to predefined qualitative categories. Particles displaying more than one clearly distinguishable colour without a predominant colour were classified as multicoloured. Background contamination was monitored using reagent, digestion, procedural, and air-exposure blanks. Blank filters were examined using the same microscopic protocol applied to clinical samples. Any particles detected in blank controls were documented and compared with those identified in clinical specimens to ensure that reported MPs were not attributable to laboratory contamination.

### 2.3. Raman Spectroscopic Characterisation

**Figure 1:**
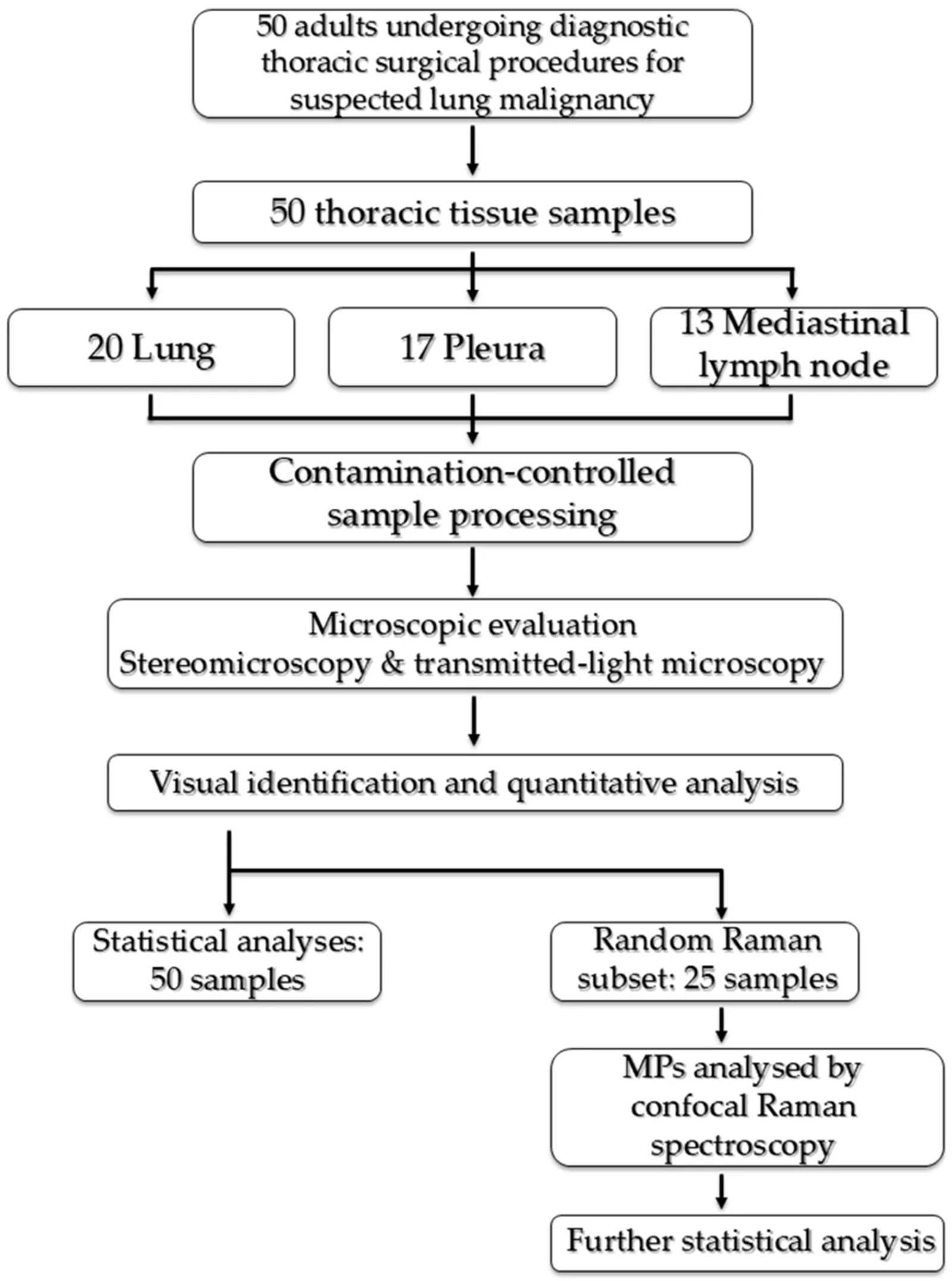
Overview of patient enrolment, thoracic tissue sampling, contamination-controlled laboratory processing, microscopic particle evaluation, Raman spectroscopic confirmation, and statistical analysis.

Following stereomicroscopic and transmitted-light microscopic evaluation, spectroscopic characterisation was performed on a randomly selected subset of 25 tissue specimens (50%). Tissue specimens were assigned numerical identifiers (1–50), and 25 specimens were selected using a computer-generated random number generator. All MPs identified within these specimens (31 of the 62 particles identified overall; 50%) subsequently underwent Raman spectroscopic analysis. Random selection of tissue specimens minimised selection bias while maintaining analytical feasibility, consistent with published recommendations for representative subsampling in microplastic research [24]. Each tissue specimen had an equal probability of selection, irrespective of diagnosis, biopsy site, or particle burden. Non-destructive Raman characterisation was performed directly on the cellulose nitrate membrane filters without further sample preparation using a Renishaw inVia Reflex confocal micro-Raman spectrometer (Renishaw plc, Wotton-under-Edge, Gloucestershire, UK) at the School of Mining and Metallurgical Engineering, National Technical University of Athens, Greece. Spectra were acquired at room temperature using both 532- and 785-nm excitation wavelengths over a spectral range of 200–3500 cm^−1^. The Raman-scattered light was dispersed using a diffraction grating with 1800 lines/mm. The laser beam was focused through a Leica microscope using a 100× short-working-distance objective, producing a spot diameter of approximately 1 μm and a power of approximately 4 mW at the sample. Exposure times of 10–20 s and one or two accumulation cycles were used depending on the material and signal quality. Wavenumber calibration was routinely verified using the silicon longitudinal optical phonon peak at 521 cm^−1^. Spectra were processed using WiRE 3.4 and SpectraGryph 1.2. Peaks were assigned by comparison with published reference spectra [25,26]. Polymer confirmation required concordance with characteristic Raman bands of a recognised polymer. Spectra showing identifiable synthetic pigment bands but insufficient polymer-specific bands were classified as pigment-dominated, whereas spectra without reliably assignable Raman bands because of persistent fluorescence were classified as fluorescence-only. The synthetic-anthropogenic category comprised polymer-confirmed and pigment-dominated spectra. Particles yielding fluorescence-only spectra were retained in the total particle counts because inclusion was determined before Raman analysis using the combined stereomicroscopic and transmitted-light microscopic criteria. Fluorescence-only indicated that assignable Raman bands could not be obtained under the applied acquisition conditions rather than a negative identification; these particles were reported separately and were not assigned a polymer identity. The overall study workflow is illustrated in Figure 1.

### 2.4. Statistical Analysis

The distribution of continuous variables was assessed using the Shapiro-Wilk test. Continuous variables are presented as median (interquartile range; range), and categorical variables as number and percentage. Comparisons between two independent groups were performed using the Mann-Whitney U test for continuous variables and Pearson’s χ^2^ test or Fisher’s exact test for categorical variables, as appropriate. Comparisons across more than two groups were performed using the Kruskal-Wallis test, followed by Bonferroni-adjusted pairwise comparisons where applicable. Associations between continuous or ordinal variables were assessed using Spearman’s rank correlation coefficient. Effect sizes for Mann-Whitney comparisons were calculated as r = Z/√N. Multivariable binary logistic regression was used to assess factors independently associated with tissue microplastic detection, adjusting for age, cumulative smoking exposure (pack-years), and primary lung malignancy. Age and smoking exposure were entered into the logistic regression model as continuous variables. Results are reported as odds ratios with 95% confidence intervals. Model calibration was assessed using the Hosmer-Lemeshow goodness-of-fit test and explanatory performance using Nagelkerke’s R^2^. Statistical analyses were performed using IBM SPSS Statistics for Windows, version 20.0 (IBM Corp., Armonk, NY, USA). All statistical tests were two-sided, and *p* < 0.05 was considered statistically significant.

## 3. Results

### 3.1. Study Population

A total of 50 thoracic tissue specimens from 50 patients undergoing diagnostic thoracic surgical procedures were included (Table 1). The median age was 63.5 years (IQR, 21; range, 36–80 years). Thirty-eight patients (76%) were male; 13 (26%) were never-smokers, 18 (36%) former smokers, and 19 (38%) current smokers, with a median smoking exposure of 46.6 pack-years (IQR, 40; range, 15–100). Tissue specimens were obtained from the lung (n = 20, 40%), pleura (n = 17, 34%), and mediastinal lymph nodes (n = 13, 26%). Overall, 32 patients (64%) were diagnosed with malignancy, comprising 22 patients with primary lung malignancy and 10 with metastatic malignancy, whereas 18 patients (36%) had benign diagnoses. Primary pulmonary malignancies comprised primary pulmonary carcinomas (adenocarcinoma, n = 9; squamous cell carcinoma, n = 1; small cell lung cancer, n = 2) and primary pulmonary lymphoma (n = 10). The final diagnoses of patients with benign disease and metastatic malignancy are summarised in Supplementary Table S1.

**Figure 2:**
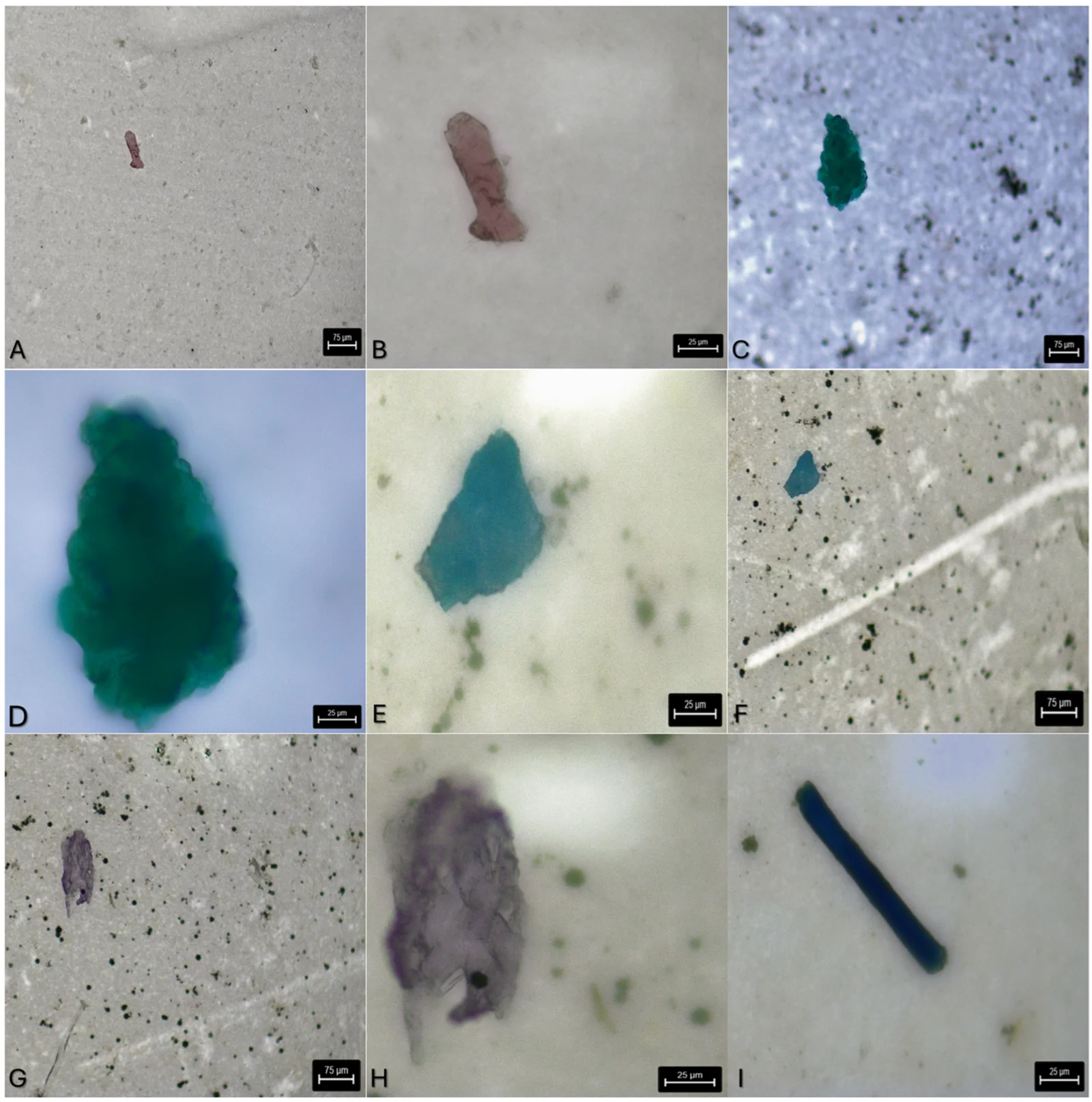
Representative stereomicroscopic and transmitted-light microscopic images of microplastic particles identified in thoracic tissue specimens. (**A**,**B**) Red fragment identified in a pleural biopsy specimen from a patient with a benign diagnosis at ×100 and ×400 magnification, respectively. (**C**,**D**) Turquoise fragment identified in a mediastinal lymph node biopsy specimen from a patient with a benign diagnosis at ×100 and ×400 magnification, respectively. (**E**,**F**) Blue fragment identified in a lung biopsy specimen from a patient with primary lung malignancy at ×100 and ×400 magnification, respectively. (**G**,**H**) Purple fragment identified in a lung biopsy specimen from a patient with primary lung malignancy at ×100 and ×400 magnification, respectively. (**I**) Blue fibre identified in a pleural biopsy specimen from a patient with a benign diagnosis at ×400 magnification. Scale bars are shown in each panel.

Low-level background contamination (0.1 particle/blank) consisted predominantly of isolated fibres distinct from clinical particles and did not affect particle classification or quantitative analyses. Across the tissue specimens, 62 MPs were identified (Table 2) comprising 60 fragments (97%) and two fibres (3%). MPs were detected in 32/50 patients (64%), including 15/20 lung specimens (75%), 8/17 pleural specimens (47%), and 9/13 mediastinal lymph node specimens (69%). The median MP burden was one particle per specimen (IQR, 2; range, 0–5). Median Dmax was 77.89 μm (IQR, 99.81; range, 22.53–636.36), and median DFeretmin was 25.54 μm (IQR, 42.91; range, 4.78–242.28). Blue particles predominated (33/62, 53%), followed by red (9/62, 15%) and orange particles (8/62, 13%); each remaining colour category accounted for <10% of identified particles. Detailed particle-level morphological characteristics and Raman spectroscopic findings for all 62 identified MPs are provided in Supplementary Table S2. Representative examples of MPs identified in thoracic tissue specimens are shown in Figure 2.

### 3.2. Microplastic Detection and Burden According to Lung Malignancy

**Figure 3:**
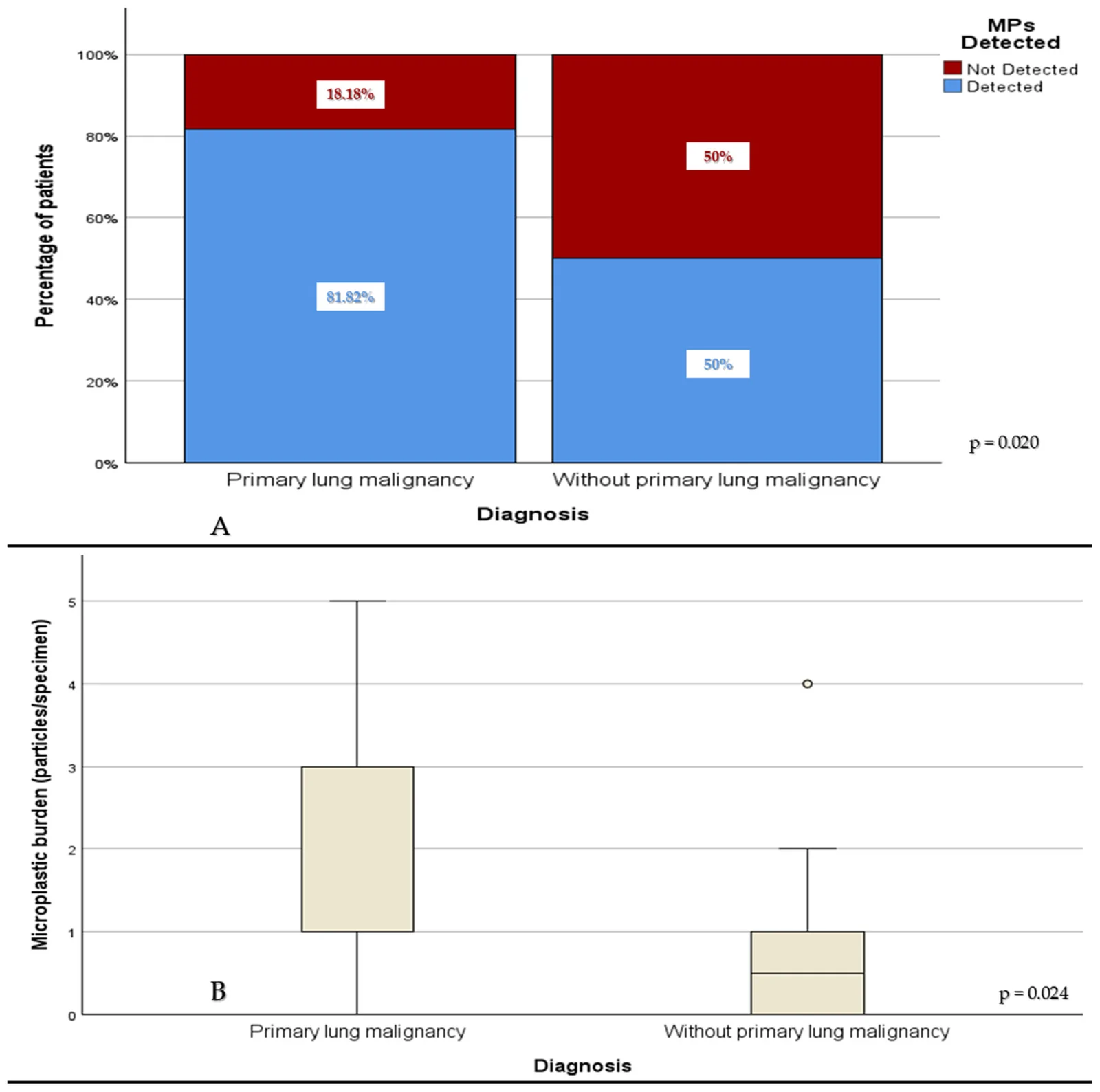
Tissue microplastic detection and burden according to primary lung malignancy. (**A**) Microplastics were detected significantly more frequently in patients with primary lung malignancy than in those without primary lung malignancy (*p* = 0.020). (**B**) Patients with primary lung malignancy had a significantly higher tissue microplastic burden than patients without primary lung malignancy (*p* = 0.024).

Microplastic detection was significantly more frequent in patients with primary lung malignancy than in those without primary lung malignancy (18/22 [81.82%] vs. 14/28 [50.0%], χ^2^(1) = 5.41, *p* = 0.020; Figure 3A). Similarly, patients with primary lung malignancy had a significantly higher microplastic burden than those without primary lung malignancy (*p* = 0.024; Figure 3B).

In multivariable logistic regression adjusting for age, smoking exposure, and primary lung malignancy, increasing age remained independently associated with a lower likelihood of microplastic detection (OR 0.93 per year, 95% CI 0.88–0.99; *p* = 0.017), whereas primary lung malignancy remained independently associated with higher odds of microplastic detection (OR 4.09, 95% CI 1.06–15.79; *p* = 0.041). Smoking exposure was not independently associated with microplastic detection (OR 1.02 per pack-year, 95% CI 0.99–1.04; *p* = 0.131) in the multivariable model. The multivariable model demonstrated good calibration (Hosmer-Lemeshow goodness-of-fit test, *p* = 0.168) and moderate explanatory performance (Nagelkerke R^2^ = 0.318). The complete regression model is presented in Supplementary Table S3.

Exploratory analyses comparing three diagnostic groups (primary lung malignancy, metastatic malignancy, and benign disease) demonstrated a significant overall difference in MP detection (χ^2^ = 6.09, *p* = 0.048). MP detection was highest in patients with primary lung malignancy (81.8%), followed by benign disease (55.6%) and metastatic malignancy (40.0%). A similar trend was observed for tissue MP burden across the three groups, although the overall difference did not reach statistical significance (Kruskal-Wallis *p* = 0.061).

### 3.3. Clinicopathological Correlations of Microplastic Detection and Burden

Exploratory analyses were subsequently performed to investigate associations between tissue MP detection and routinely collected demographic, clinicopathological, and laboratory parameters. Patients with detectable MPs were significantly younger (*p* = 0.023) and had lower median mean corpuscular volume (*p* = 0.019), platelet count (*p* = 0.020), and serum albumin (*p* = 0.006). Detection rates did not differ according to smoking status, pack-years (*p* = 0.275), biopsy site, overall malignancy status, or lung malignancy subtype (*p* = 0.252). No other laboratory parameters differed significantly between patients with and without detectable MPs.

Microplastic burden also differed according to sex, with males exhibiting a higher burden than females (*p* = 0.015). No significant differences were observed according to smoking status, biopsy site, overall malignancy status, or lung malignancy subtype.

Spearman correlation analysis demonstrated a moderate inverse correlation between microplastic burden and age (ρ = −0.484, *p* < 0.001). Higher microplastic burden was also associated with lower MCV (ρ = −0.343, *p* = 0.015), whereas positive correlations were observed with platelet count (ρ = 0.368, *p* = 0.009) and serum albumin (ρ = 0.364, *p* = 0.009). A weak inverse correlation was observed with lactate dehydrogenase (ρ = −0.327, *p* = 0.021). No other laboratory parameters correlated significantly with microplastic burden.

Within the primary lung malignancy subgroup, microplastic burden demonstrated strong positive correlations with neutrophil-to-lymphocyte ratio (ρ = 0.835, *p* < 0.001) and platelet count (ρ = 0.439, *p* = 0.041), and strong inverse correlations with lactate dehydrogenase (ρ = −0.833, *p* < 0.001), lymphocyte count (ρ = −0.720, *p* < 0.001), monocyte count (ρ = −0.669, *p* < 0.001), basophil count (ρ = −0.531, *p* = 0.011), and MCV (ρ = −0.470, *p* = 0.027). No additional laboratory parameters correlated significantly with microplastic burden within the primary lung malignancy subgroup.

### 3.4. Morphological Characteristics of Identified Microplastics

**Figure 4:**
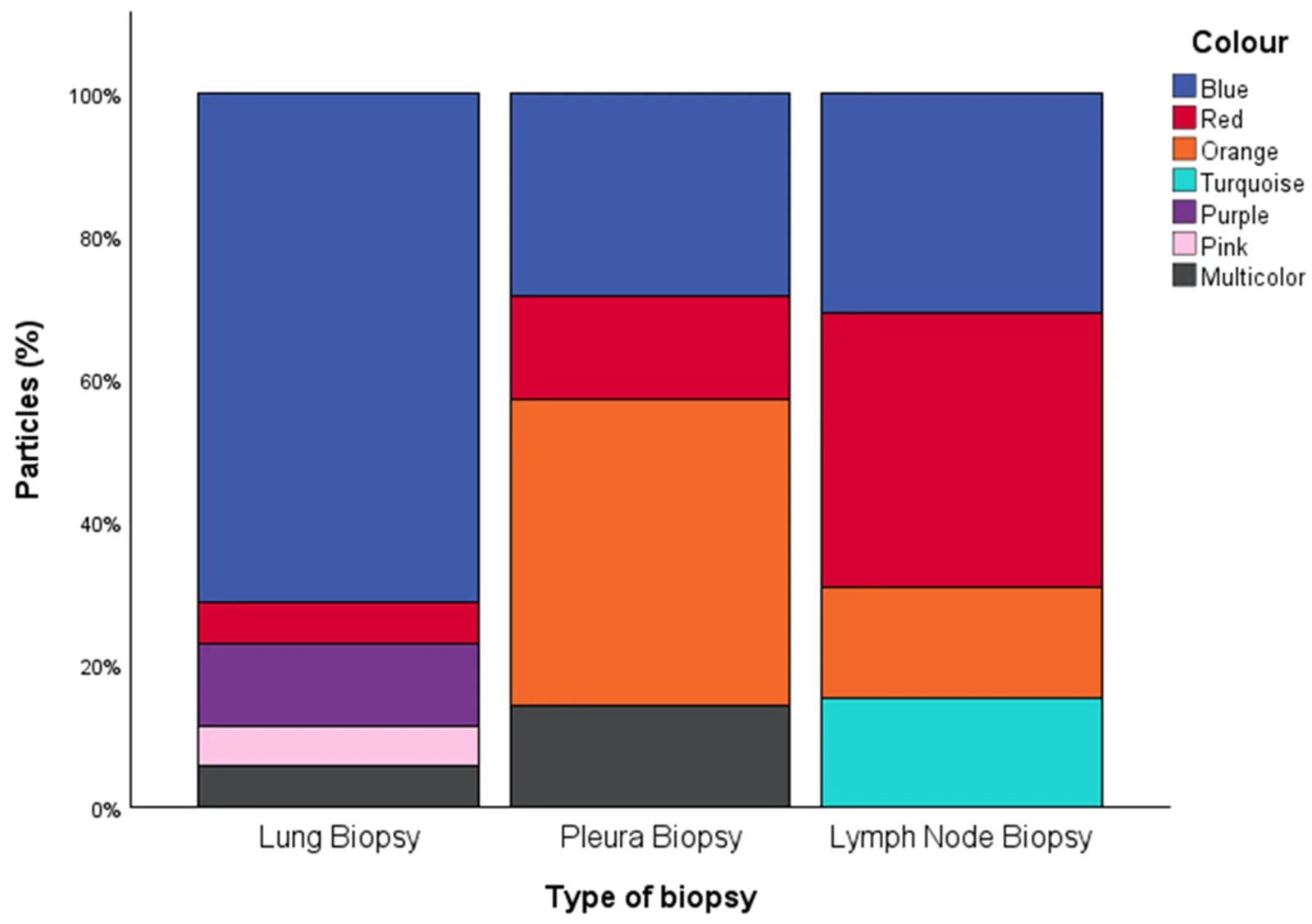
Distribution of microplastic particle colours according to biopsy site. One hundred percent stacked bar chart showing the relative distribution of particle colours in lung, pleural, and mediastinal lymph node biopsy specimens.

Particle morphology differed primarily according to biopsy site rather than malignancy status. Across the overall cohort, particle colour distribution varied according to biopsy site (Cramer’s V = 0.573, *p* < 0.001; Figure 4), with blue particles predominating in lung biopsy specimens, orange particles in pleural biopsy specimens, and red particles in lymph node biopsy specimens. The method of particle identification also varied according to biopsy site (Cramer’s V = 0.378, *p* = 0.007); particles with synthetic-anthropogenic spectral signatures were identified in both lung and pleural biopsy specimens, whereas particles yielding fluorescence-only spectra were observed exclusively in lung biopsy specimens. Similar findings were observed in malignant cases (Cramer’s V = 0.694, *p* < 0.001) and became more pronounced in the primary lung malignancy subgroup (Cramer’s V = 0.832, *p* < 0.001). Particle colour distribution was not associated with overall malignancy status or primary lung malignancy.

### 3.5. Particle Size Characteristics

**Figure 5:**
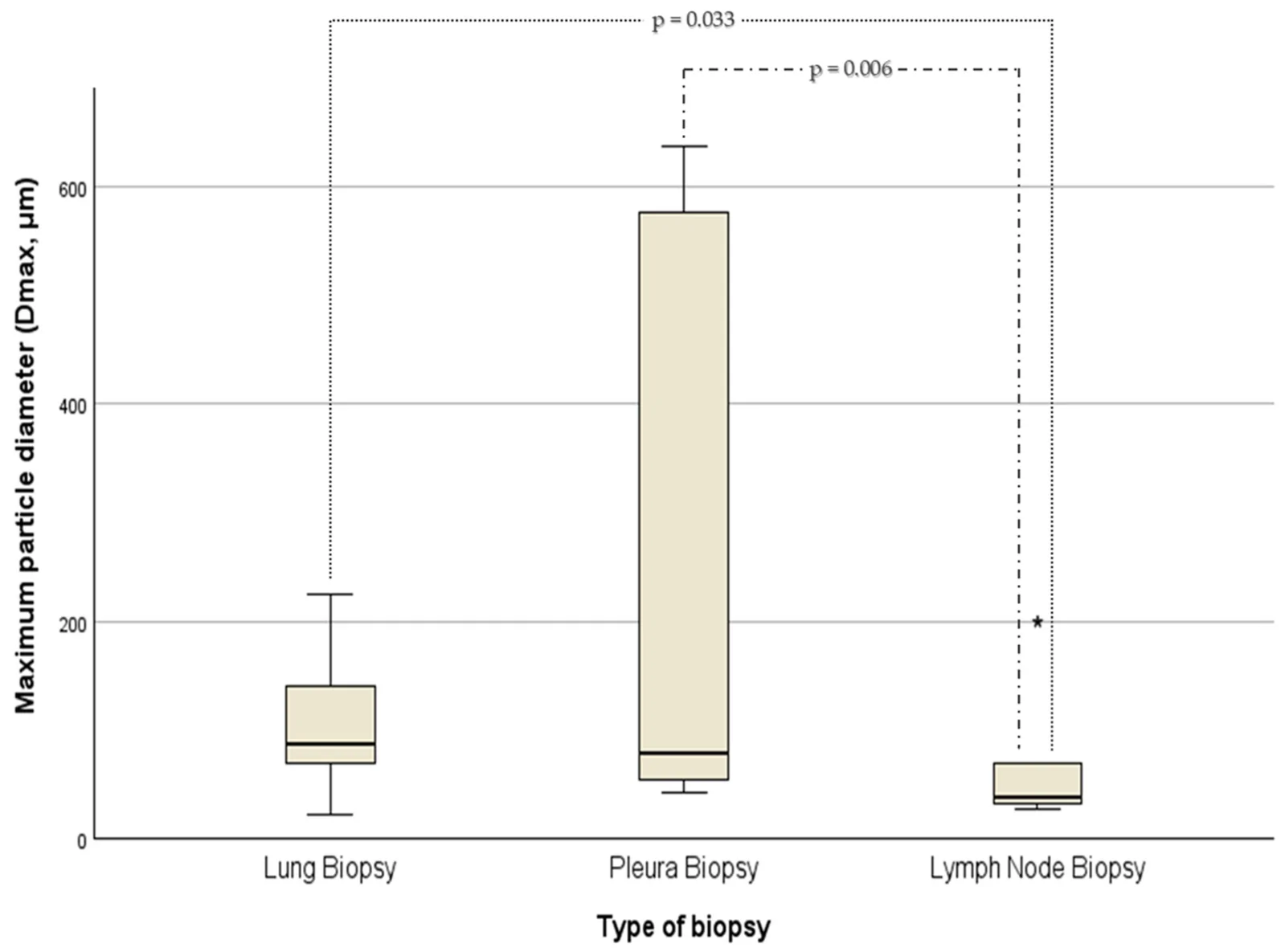
Maximum microplastic particle diameter according to biopsy site. Boxplots showing the distribution of maximum particle diameter (Dmax) in lung, pleural, and mediastinal lymph node biopsy specimens. Dmax differed significantly according to biopsy site, with smaller particles identified in lymph node biopsy specimens.

Particle size analyses demonstrated moderate agreement between Dmax and DFeretmin (ρ = 0.480, *p* < 0.001). Dmax differed according to several particle characteristics, whereas DFeretmin remained consistent across all comparisons. Particles yielding fluorescence-only spectra exhibited significantly greater Dmax values than particles with synthetic-anthropogenic spectral signatures (*p* = 0.017, r = 0.43). Dmax also varied according to biopsy site (*p* = 0.006; Figure 5); Bonferroni-adjusted post hoc analysis demonstrated significantly lower Dmax values in lymph node biopsy specimens than in both lung (adjusted *p* = 0.033) and pleural biopsy specimens (adjusted *p* = 0.006), whereas lung and pleural biopsy specimens did not differ (adjusted *p* = 0.726). Dmax also differed across particle colour groups (*p* = 0.013), with post hoc analysis identifying a significant difference only between orange and red particles (adjusted *p* = 0.012). Neither Dmax nor DFeretmin differed according to malignancy status or malignancy subtype, and DFeretmin showed no significant associations with biopsy site or particle colour.

## 4. Discussion

This prospective study suggests that MPs are frequently present within diagnostically obtained thoracic tissues and are more commonly detected in patients with primary lung malignancy than in patients without primary lung malignancy. Furthermore, particle morphology and size differed according to biopsy site rather than malignancy status, suggesting that respiratory MP accumulation is compartment-specific within the thorax. Together, these findings extend current knowledge of human respiratory MP distribution while providing clinically relevant evidence from a prospectively recruited diagnostic surgical cohort studied under rigorous contamination-control conditions.

Our findings are consistent with, while substantially extending, previous human studies investigating respiratory MPs. Initial evidence of pulmonary MP deposition was provided by Amato-Lourenço et al., who demonstrated airborne MPs within surgically resected human lung tissue, followed by Jenner et al., who independently confirmed pulmonary MP accumulation using micro-Fourier transform infrared spectrometry [5,6]. More recently, MPs have also been identified in BAL fluid obtained from living individuals, including never-smokers, providing compelling evidence that inhaled particles reach the distal respiratory tract under physiological conditions [7,8,9,10]. Similarly, Zhao et al. detected MPs across several human tumour types, whereas Gholami et al. recently demonstrated distinct MP signatures between malignant and adjacent non-malignant lung tissues [11,13]. Our study complements these observations by examining multiple thoracic tissue compartments obtained during routine diagnostic procedures rather than surgical cancer resections alone. Moreover, by including a contemporaneous comparison group of patients without primary lung malignancy and demonstrating that primary lung malignancy remained independently associated with increased tissue MP detection after adjustment for smoking exposure and age, our findings provide additional evidence that respiratory tissue MP accumulation is more closely associated with primary lung malignancy than with malignancy in general while appropriately avoiding causal inferences.

Exploratory analyses separating metastatic malignancy from benign disease demonstrated that the overall difference in MP detection across diagnostic categories was primarily attributable to patients with primary lung malignancy. Although these subgroup analyses were limited by the relatively small number of metastatic cases and should therefore be interpreted cautiously, they further support our primary hypothesis that the observed association is specific to primary lung malignancy rather than reflecting malignancy in general.

Beyond demonstrating the presence of MPs in thoracic tissues, our findings emphasise the importance of evaluating multiple thoracic tissue compartments. Previous investigations have largely focused on single specimen types, including surgically resected lung tissue, BAL fluid, or isolated tumour samples [5,6,7,8,9,10,11,12,13]. While these studies have established that inhaled MPs reach the human respiratory system, they provide limited information regarding how particle characteristics vary between different thoracic tissues. By including lung, pleural, and mediastinal lymph node biopsy specimens within the same prospective study, our findings suggest that respiratory MP accumulation is heterogeneous across thoracic tissue compartments, supporting the concept that different anatomical sites may exhibit distinct patterns of particle deposition, retention, and biological handling.

The higher frequency of MP detection and greater tissue burden observed in patients with primary lung malignancy warrant careful interpretation. Importantly, our findings should not be interpreted as evidence that MPs cause lung cancer. Rather, they demonstrate an association between primary lung malignancy and increased respiratory MP accumulation within thoracic tissues. Continuous atmospheric transport and deposition of airborne MPs, together with increasing evidence that inhalation represents an important route of human exposure, make chronic respiratory particle accumulation biologically plausible [3,4]. Several biological mechanisms may plausibly explain the observed association. Patients with primary lung malignancy are characterised by profound alterations in tissue architecture, extracellular matrix organisation, vascular permeability, and local immune-cell composition, all of which could influence particle deposition, retention, or clearance within the thoracic microenvironment independently of environmental exposure [12,17,21]. Conversely, chronic retention of inhaled MPs may contribute to a pro-inflammatory microenvironment characterised by oxidative stress, macrophage dysfunction, persistent immune activation, and dysregulated cellular signalling, mechanisms that have been implicated experimentally in tumour progression but remain incompletely validated in humans [14,17,18,19,20,21,22,23]. Experimental studies have also demonstrated activation of pathways involved in epithelial proliferation, ferroptosis, glutathione metabolism, and tumour immune remodelling following chronic exposure to micro- and nanoplastics [14,17,18,19,20,21]. Nevertheless, the observational nature of the present study precludes conclusions regarding temporality or causality, and it remains equally plausible that malignant tissues provide a biological niche favouring particle retention rather than representing a consequence of MP exposure.

Another noteworthy observation was that smoking exposure was not independently associated with tissue MP detection after multivariable adjustment. Although cigarette smoke contains abundant combustion-derived particulate matter, MPs originate predominantly from environmental sources of plastic particles and therefore represent a distinct class of inhaled particles [2,4]. This finding suggests that respiratory MP accumulation may reflect cumulative environmental exposure together with tissue-specific biological processes rather than smoking history alone. Nevertheless, because environmental exposure was not measured directly and the study sample was relatively small, these observations should be interpreted cautiously and confirmed in larger cohorts incorporating detailed exposure assessment.

An unexpected finding was the inverse association between age and both tissue MP detection and burden. Although older age might intuitively be considered a proxy for greater cumulative exposure, thoracic MP counts should not necessarily be interpreted as a lifetime exposure dosimeter. Human exposure to MPs varies considerably according to environmental setting, lifestyle, and age, while current knowledge regarding tissue toxicokinetics, retention, and clearance remains limited [27,28]. Consequently, tissue MP burden measured at a single time point may reflect a combination of site-specific deposition, lymphatic transport, particle clearance, tissue retention, and disease-associated trapping rather than simple cumulative exposure. A birth-cohort effect related to increasingly plastic-intensive indoor and consumer environments is also conceivable, although this was not directly assessed in the present study [27]. Therefore, a monotonic increase in thoracic MP burden with age cannot currently be assumed. Nevertheless, residual confounding, selection effects within this diagnostic cohort, and the modest sample size remain more conservative explanations. This exploratory finding should therefore be interpreted cautiously and requires confirmation in larger studies incorporating detailed lifetime exposure assessment [28,29]. Similarly, the exploratory laboratory associations should be interpreted cautiously. The differing associations observed between binary MP detection and quantitative MP burden likely reflect the exploratory nature of these analyses, the modest sample size, and the multiple comparisons performed rather than distinct biological mechanisms and therefore require confirmation in independent cohorts.

One of the principal findings of the present study was that particle characteristics were influenced predominantly by biopsy site rather than malignancy status. Although overall detection and burden were associated with primary lung malignancy, particle colour distribution and maximum particle diameter differed substantially between lung, pleural, and mediastinal lymph node specimens. To our knowledge, no previous human study has systematically compared MP characteristics across multiple thoracic tissue compartments obtained during routine diagnostic procedures. These findings suggest compartment-specific patterns of particle deposition, transport, and retention within the thorax. Experimental studies of inhaled particles have demonstrated that particle size is a major determinant of respiratory deposition and subsequent translocation, with smaller particles more readily penetrating the distal lung and entering lymphatic pathways [15,16]. The significantly smaller particles identified within mediastinal lymph nodes in our cohort are consistent with this concept and may reflect preferential lymphatic transport of smaller MPs following deposition within the pulmonary parenchyma. Similarly, the observed biopsy-site-specific differences in particle colour may indicate differences in environmental sources, polymer weathering, or tissue-specific clearance mechanisms, although these possibilities remain speculative and require confirmation in larger studies. Future studies should investigate thoracic tissues as biologically distinct compartments. The observation that particle morphology and maximum particle diameter varied according to biopsy site, whereas overall MP burden was primarily associated with primary lung malignancy, is consistent with the hypothesis that different thoracic tissues participate in distinct phases of particle handling. The pulmonary parenchyma represents the initial site of particle deposition, whereas pleural tissues may reflect secondary processes of particle redistribution or clearance. In contrast, mediastinal lymph nodes constitute an integral component of pulmonary lymphatic drainage and would be expected to preferentially accumulate smaller particles capable of lymphatic transport [15,16]. Although the biological mechanisms responsible for these compartment-specific differences remain uncertain, our findings are compatible with the hypothesis that respiratory MP accumulation may involve deposition, retention, and redistribution rather than simple passive tissue accumulation. However, because each patient contributed only a single tissue specimen, these compartment-specific observations should be regarded as hypothesis-generating and require confirmation in studies incorporating paired sampling from multiple thoracic compartments within the same individual. Elucidating these mechanisms may improve understanding of how inhaled MPs interact with the human respiratory system and may help guide future mechanistic studies investigating particle localisation, cellular uptake, and tissue-specific biological responses.

A major strength of this study is its prospective design combined with rigorous contamination-control procedures throughout sample collection and analysis. Confocal micro-Raman spectroscopy was performed on a randomly selected subset of tissue specimens, with all MPs identified within those specimens subsequently undergoing spectroscopic analysis, with more than two-thirds demonstrating synthetic anthropogenic spectral signatures. This analytical strategy is supported by recent methodological evidence demonstrating that appropriately designed random subsampling provides representative estimates of particle characteristics while substantially reducing analytical workload [24]. An additional strength of the present study is the inclusion of patients undergoing routine diagnostic thoracic procedures rather than surgical cancer resections alone. This design enabled comparison of thoracic tissues from patients with primary lung malignancy and patients without primary lung malignancy using identical contamination-control and analytical protocols, thereby reducing methodological heterogeneity. Furthermore, prospective recruitment with consecutive patient inclusion minimised selection bias and enhanced the clinical relevance of the findings. Although polymer-specific identification was not possible because of fluorescence interference, pigment signatures were identified in several particles, providing independent spectroscopic confirmation of anthropogenic material within thoracic tissues. Fluorescence remains a recognised limitation of Raman analysis in weathered environmental MPs and biological specimens, particularly when pigments, additives, or tissue-derived organic material obscure characteristic polymer bands [25,26]. Consequently, the inability to assign polymer composition to every analysed particle should not be interpreted as evidence against their anthropogenic origin but rather reflects a well-recognised analytical challenge.

Several limitations should also be acknowledged. This was a single-centre study with a relatively modest sample size, limiting statistical power for detailed subgroup analyses according to histological subtype or biopsy location. The exploratory three-group analyses should be interpreted cautiously because the metastatic subgroup was relatively small and clinically heterogeneous. Similarly, the clinicopathological and laboratory association analyses were exploratory, involved multiple comparisons, and should therefore be considered hypothesis-generating. Particle-level analyses were also exploratory and treated individual particles as the unit of analysis. Because multiple particles could originate from the same tissue specimen, within-specimen clustering may have resulted in underestimation of statistical uncertainty. Consequently, these findings should be interpreted as hypothesis-generating and require confirmation in larger studies using clustered or hierarchical analytical approaches. Microplastic burden was expressed as the number of particles detected per diagnostic tissue specimen and was not normalised to tissue weight or volume, as these measurements were not recorded before chemical digestion. The specimens comprised non-standardised diagnostic biopsy fragments from anatomically distinct thoracic compartments, selected by the attending pathologist as macroscopically representative of the sampled lesion or site rather than according to predefined research dimensions. Although simple mass-based normalisation would not fully account for the biological and procedural differences among lung, pleural, and lymph-node specimens, variation in specimen size may nevertheless have influenced particle counts and cannot be excluded. Future studies should incorporate prospective pre-digestion measurements of tissue mass or volume together with standardised compartment-specific sampling protocols. Polymer identification was incomplete because fluorescence prevented definitive spectral assignment in a proportion of analysed particles, and complementary techniques such as micro-Fourier transform infrared or pyrolysis-gas chromatography/mass spectrometry were not available. Environmental exposure could not be quantified objectively, and the cross-sectional design precludes causal inference. Finally, although thoracic tissues from multiple anatomical compartments were evaluated, the present study cannot determine the biological fate, persistence, or functional consequences of retained MPs within individual tissues. Moreover, diagnostic biopsy specimens may not fully represent the spatial heterogeneity of MP deposition throughout the entire thorax. In addition, the tissue fragment allocated for microplastic analysis was not subjected to parallel histopathological examination. Although it originated from the same diagnostic sampling procedure and was selected to represent the sampled lesion, the precise microscopic composition of the analysed fragment could not be independently confirmed. Longitudinal sampling was also not feasible, precluding assessment of temporal changes in particle accumulation and clearance.

## 5. Conclusions

Microplastics were frequently detected in diagnostically obtained thoracic tissue specimens despite rigorous contamination-control procedures, providing further evidence that particles accumulate within the human respiratory system. Patients with primary lung malignancy exhibited significantly greater tissue microplastic detection and burden than patients without primary lung malignancy, whereas compartment-specific particle characteristics suggested distinct patterns of respiratory micro-plastic deposition and retention. Exploratory analyses further indicated that this association was more closely related to primary lung malignancy than to malignancy in general. Although these findings do not establish a causal relationship between micro-plastics and lung cancer, they suggest that disease status and tissue-specific biological characteristics may influence particle accumulation. This prospective study extends current knowledge of respiratory microplastic distribution in humans and supports the concept that thoracic tissues should be considered biologically distinct compartments when investigating respiratory microplastic accumulation. Future multicentre studies integrating detailed exposure assessment, advanced spectroscopic techniques, and molecular tissue profiling are required to clarify the mechanisms governing tissue retention and their potential clinical significance.

## Figures and Tables

**Table 1:** Baseline demographic, clinical, and microplastic burden characteristics of the study population (n = 50).

Variable	n (%) or Median (IQR; Min–Max)
Age (years)	63.5 (21; 36–80)
Gender (Male/Female)	38 (76%)/12 (24%)
Smoking status (Non/Ex/Active)	13 (26%)/18 (36%)/19 (38%)
Pack-years	46.6 (40; 15–100)
Tissue specimen (Lung/Pleura/Lymph Nodes)	20 (40%)/17 (34%)/13 (26%)
Diagnosis of cancer (No/Yes)	18 (36%)/32 (64%)
Primary lung malignancy among malignant cases (No/Yes)	10 (31%)/22 (69%)
Histological subtype of primary lung malignancy cases [n = 22]	
Pulmonary Carcinoma (adenocarcinoma/squamous cell carcinoma/small-cell carcinoma)	9 (41%)/1 (5%)/2 (9%)
Primary pulmonary lymphoma	10 (45%)
Microplastics presence in patients (No/Yes) [n = 50]	18 (36%)/32 (64%)
Microplastics presence in lung biopsies (No/Yes) [n = 20]	5 (25%)/15 (75%)
Microplastics presence in pleura biopsies (No/Yes) [n = 17]	9 (53%)/8 (47%)
Microplastics presence in lymph nodes biopsies (No/Yes) [n = 13]	4 (31%)/9 (69%)
Total Microplastics (Fragments/Fibers)	62 (60–97%/2–3%)
Lung biopsies Microplastics (Fragments/Fibers)	35 (33–94%/2–6%)
Pleura biopsies Microplastics (Fragments/Fibers)	14 (14–100%/0–0%)
Lymph node biopsies Microplastics (Fragments/Fibers)	13 (13–100%/0–0%)

**Table 2:** Distribution of particle-level microplastic morphological, colour, and Raman spectroscopic characteristics according to diagnostic category. All data in this table are particle-based. Percentages were calculated using the total number of particles in each column as the denominator (overall, n = 62; benign disease, n = 18; malignancy, n = 44; primary lung malignancy, n = 38).

Characteristic	Overall	Benign Disease	Malignancy	Lung Malignancy
Total microplastics	62	18	44	38
Median Dmax μm (IQR; range)	77.89 (99.81; 22.53–636.36)	69.87 (138.11; 22.53–224.49)	79.24 (97.97; 27.06–636.36)	80.31 (98.57; 27.06–636.36)
Median DFeretmin μm (IQR; range)	25.54 (42.91; 4.78–242.28)	24.78 (25.50; 4.78–125.05)	26.28 (42.05; 12.75–242.28)	27.63 (50.47; 12.75–242.28)
Fragments (%)	60 (97%)	16 (89%)	44 (100%)	38 (100%)
Fibres (%)	2 (3%)	2 (11%)	0 (0%)	0 (0%)
Blue (%)	33 (53%)	10 (56%)	23 (52%)	19 (50%)
Red (%)	9 (15%)	2 (11%)	7 (16%)	5 (13%)
Orange (%)	8 (13%)	2 (11%)	6 (14%)	6 (16%)
Multicolour (%)	4 (6.5%)	2 (11%)	2 (4.5%)	2 (5%)
Purple (%)	4 (6.5%)	0 (0%)	4 (9%)	4 (11%)
Pink (%)	2 (3%)	0 (0%)	2 (4.5%)	2 (5%)
Turquoise (%)	2 (3%)	2 (11%)	0 (0%)	0 (0%)
**Raman Subset Particles**	**n = 31/62**	**n = 12/18**	**n = 19/44**	**n = 13/38**
Synthetic-anthropogenic spectral signatures (%)	22 (71%)	8 (67%)	14 (74%)	8 (62%)
Fluorescence-only spectra (%)	9 (29%)	4 (33%)	5 (26%)	5 (38%)

## Data Availability

The original contributions presented in this study are included in the article. Further inquiries can be directed to the corresponding author.

## Supplementary Materials

The following supporting information can be downloaded at: https://www.mdpi.com/article/10.3390/jcm15176503/s1, Table S1: Final diagnoses of patients with benign disease (n = 18) and metastatic malignancy (n = 10) included in the study. All participants underwent diagnostic thoracic tissue sampling for suspected primary lung malignancy; the diagnoses listed represent the final diagnosis established following routine histopathological evaluation; Table S2: Particle-level morphological and Raman spectroscopic characteristics of all microplastics detected in thoracic tissue specimens (n = 62). Confocal micro-Raman spectroscopy was performed on all particles identified within a randomly selected subset of 25 tissue specimens (31/62 particles). The table includes the study group, tissue compartment, particle morphology, colour, particle dimensions (Dmax and DFeretmin), Raman analysis status, and spectral classification. Fields relating to Raman analysis are reported as “Not analysed” for particles that were not selected for spectroscopic characterisation. Participant IDs represent anonymised study identifiers and are therefore not sequential; Table S3: Multivariable logistic regression model evaluating factors independently associated with microplastic detection in thoracic tissue specimens. Age and smoking exposure were entered as continuous variables, and primary lung malignancy was entered as a binary categorical variable. Model fit statistics were as follows: omnibus test of model coefficients χ^2^ = 13.18 (df = 3, *p* = 0.004), Nagelkerke R^2^ = 0.318, and Hosmer-Lemeshow goodness-of-fit test *p* = 0.168. Overall classification accuracy was 72.0%.

## Abbreviations

The following abbreviations are used in this manuscript:

BAL	bronchoalveolar lavage
DFeretmin	Feret’s minimum diameter
Dmax	maximum diameter
IQR	interquartile range
MCV	mean corpuscular volume
MPs	microplastics
STROBE	Strengthening the Reporting of Observational Studies in Epidemiology
