# Supplementary material for: Microplastics in Human Thoracic Tissues: Increased Burden in Patients with Lung Malignancy
Source: J Clin Med. 2026 Aug 22;15(17):6503. doi: 10.3390/jcm15176503 (PMC13565977; doi:10.3390/jcm15176503)
Supplement: Supplementary file 1 [file jcm-15-06503-s001.zip › jcm-4458187-supplementary.pdf]

Supplementary Tables

Table S1: *Final diagnoses of patients with benign disease (n = 18) and metastatic malignancy (n = 10) included in the study. All participants underwent diagnostic thoracic tissue sampling for suspected primary lung malignancy; the diagnoses listed represent the final diagnosis established following routine histopathological evaluation.*

Participant ID	Category	Final diagnosis
01	Benign Disease	Chronic non-specific pleuritis
03	Benign Disease	Sarcoidosis
04	Metastatic Malignancy	Metastatic renal cell carcinoma
05	Benign Disease	Chronic non-specific pleuritis
06	Benign Disease	Sarcoidosis
09	Benign Disease	Sarcoidosis
10	Metastatic Malignancy	Metastatic renal cell carcinoma
11	Benign Disease	Chronic non-specific pleuritis
12	Benign Disease	Chronic non-specific pleuritis
13	Metastatic Malignancy	Metastatic breast carcinoma
15	Metastatic Malignancy	Metastatic colorectal adenocarcinoma

16	Metastatic Malignancy	Metastatic breast carcinoma
17	Metastatic Malignancy	Metastatic breast carcinoma
19	Benign Disease	Tuberculosis
20	Benign Disease	Tuberculosis
22	Metastatic Malignancy	Metastatic breast carcinoma
24	Benign Disease	Necrotising granulomatous inflammation
25	Metastatic Malignancy	Metastatic sarcoma
26	Benign Disease	Rheumatoid Nodule
27	Benign Disease	Chronic pleuritis associated with rheumatoid arthritis
29	Benign Disease	Sarcoidosis
30	Benign Disease	Fungal infection
32	Metastatic Malignancy	Metastatic colorectal adenocarcinoma
34	Benign Disease	Inflammatory pseudotumour

36	Metastatic Malignancy	Metastatic colorectal adenocarcinoma
38	Benign Disease	Cryptogenic organising pneumonia
45	Benign Disease	Cryptogenic organising pneumonia
48	Benign Disease	Cryptogenic organising pneumonia

Table S2: *Particle-level morphological and Raman spectroscopic characteristics of all microplastics detected in thoracic tissue specimens (n = 62). Confocal micro-Raman spectroscopy was performed on all particles identified within a randomly selected subset of 25 tissue specimens (31/62 particles). The table includes the study group, tissue compartment, particle morphology, colour, particle dimensions (Dmax and DFeretmin), Raman analysis status, and spectral classification. Fields relating to Raman analysis are reported as "Not analysed" for particles that were not selected for spectroscopic characterisation. Participant IDs represent anonymised study identifiers and are therefore not sequential.*

Particle ID	Participant ID	Study group	Sample compartment	Morphology	Colour	Dmax (µm)	DFeretmin (µm)	Raman analysis status	Spectral classification
P001	02	Lung Malignancy	Lung	Fragment	Blue	33,81	24,8	Analysed	Synthetic/Anthropogenic
P002	02	Lung Malignancy	Lung	Fragment	Blue	33,82	24,9	Analysed	Synthetic/Anthropogenic
P003	03	Benign Disease	Lymph Node	Fragment	Blue	38,82	24,79	Not Analysed	Not Analysed
P004	03	Benign Disease	Lymph Node	Fragment	Orange	69,88	24,78	Not Analysed	Not Analysed
P005	06	Benign Disease	Lung	Fibre	Blue	224,5	4,78	Analysed	Fluorescence only

P006	06	Benign Disease	Lung	Fragment	Red	113,57	43,49	Analysed	Fluorescence only
P007	06	Benign Disease	Lung	Fragment	Blue	22,52	14,44	Analysed	Synthetic/Anthropogenic
P008	06	Benign Disease	Lung	Fragment	Blue	27,83	16,25	Analysed	Synthetic/Anthropogenic
P009	07	Lung Malignancy	Lung	Fragment	Purple	84,09	26,28	Analysed	Fluorescence only
P010	07	Lung Malignancy	Lung	Fragment	Blue	72,19	71,05	Analysed	Synthetic/Anthropogenic
P011	07	Lung Malignancy	Lung	Fragment	Pink	75,09	28,99	Analysed	Fluorescence only
P012	07	Lung Malignancy	Lung	Fragment	Multicolour	141,5	21,88	Analysed	Synthetic/Anthropogenic
P013	08	Lung Malignancy	Lymph Node	Fragment	Blue	39,82	23,87	Not Analysed	Not Analysed
P014	08	Lung Malignancy	Lymph Node	Fragment	Blue	39,81	23,86	Not Analysed	Not Analysed
P015	09	Benign Disease	Pleura	Fragment	Multicolour	54,09	40,58	Analysed	Synthetic/Anthropogenic
P016	09	Benign Disease	Pleura	Fragment	Multicolour	54,08	40,57	Analysed	Synthetic/Anthropogenic

P017	14	Lung Malignancy	Pleura	Fragment	Orange	636,3	14,3	Not Analysed	Not Analysed
P018	14	Lung Malignancy	Pleura	Fragment	Orange	575,3	242,2	Not Analysed	Not Analysed
P019	14	Lung Malignancy	Pleura	Fragment	Orange	295,26	140,57	Not Analysed	Not Analysed
P020	15	Metastatic Malignancy	Lung	Fragment	Blue	86,66	59,08	Analysed	Synthetic/Anthropogenic
P021	15	Metastatic Malignancy	Lung	Fragment	Blue	86,65	59,07	Analysed	Synthetic/Anthropogenic
P022	16	Metastatic Malignancy	Pleura	Fragment	Red	79,24	24,38	Analysed	Synthetic/Anthropogenic
P023	16	Metastatic Malignancy	Pleura	Fragment	Blue	42,19	24,38	Analysed	Synthetic/Anthropogenic
P024	16	Metastatic Malignancy	Pleura	Fragment	Red	79,23	24,37	Analysed	Synthetic/Anthropogenic
P025	16	Metastatic Malignancy	Pleura	Fragment	Blue	42,18	24,37	Analysed	Synthetic/Anthropogenic
P026	18	Lung Malignancy	Pleura	Fragment	Blue	76,55	55,05	Analysed	Synthetic/Anthropogenic
P027	18	Lung Malignancy	Pleura	Fragment	Blue	76,54	55,04	Analysed	Synthetic/Anthropogenic

P028	21	Lung Malignancy	Lymph Node	Fragment	Red	32,07	19,95	Not Analysed	Not Analysed
P029	21	Lung Malignancy	Lymph Node	Fragment	Red	27,06	16,8	Not Analysed	Not Analysed
P030	23	Lung Malignancy	Lung	Fragment	Blue	98,75	70,06	Not Analysed	Not Analysed
P031	23	Lung Malignancy	Lung	Fragment	Blue	70,04	14,69	Not Analysed	Not Analysed
P032	28	Lung Malignancy	Lung	Fragment	Purple	43,85	12,75	Not Analysed	Not Analysed
P033	28	Lung Malignancy	Lung	Fragment	Purple	43,84	12,74	Not Analysed	Not Analysed
P034	29	Benign Disease	Lung	Fragment	Blue	165,77	39,56	Analysed	Synthetic/Anthropogenic
P035	29	Benign Disease	Lung	Fragment	Blue	165,76	39,55	Analysed	Synthetic/Anthropogenic
P036	31	Lung Malignancy	Lymph Node	Fragment	Red	29,56	18,43	Not Analysed	Not Analysed
P037	33	Lung Malignancy	Lung	Fragment	Blue	140,57	62,5	Not Analysed	Not Analysed
P038	33	Lung Malignancy	Lung	Fragment	Blue	161,05	62,5	Not Analysed	Not Analysed

P039	33	Lung Malignancy	Lung	Fragment	Blue	102,29	71,62	Not Analysed	Not Analysed
P040	35	Lung Malignancy	Lung	Fragment	Purple	84,08	26,27	Analysed	Fluorescence only
P041	35	Lung Malignancy	Lung	Fragment	Blue	72,18	71,04	Analysed	Synthetic/Anthropogenic
P042	35	Lung Malignancy	Lung	Fragment	Pink	75,08	28,98	Analysed	Fluorescence only
P043	35	Lung Malignancy	Lung	Fragment	Multicolour	141,4	21,87	Analysed	Synthetic/Anthropogenic
P044	37	Lung Malignancy	Lung	Fragment	Blue	98,76	70,05	Not Analysed	Not Analysed
P045	37	Lung Malignancy	Lung	Fragment	Blue	70,05	14,7	Not Analysed	Not Analysed
P046	38	Benign Disease	Lung	Fibre	Blue	224,49	4,77	Analysed	Fluorescence only
P047	38	Benign Disease	Lung	Fragment	Red	113,56	43,48	Analysed	Fluorescence only
P048	38	Benign Disease	Lung	Fragment	Blue	22,53	14,43	Analysed	Synthetic/Anthropogenic
P049	38	Benign Disease	Lung	Fragment	Blue	27,82	16,24	Analysed	Synthetic/Anthropogenic

P050	41	Lung Malignancy	Lung	Fragment	Blue	123,07	50,48	Analysed	Fluorescence only
P051	43	Lung Malignancy	Lung	Fragment	Blue	140,56	62,49	Not Analysed	Not Analysed
P052	43	Lung Malignancy	Lung	Fragment	Blue	161,04	62,49	Not Analysed	Not Analysed
P053	43	Lung Malignancy	Lung	Fragment	Blue	102,28	71,61	Not Analysed	Not Analysed
P054	45	Benign Disease	Lymph Node	Fragment	Turquoise	199,43	125,06	Not Analysed	Not Analysed
P055	45	Benign Disease	Lymph Node	Fragment	Turquoise	199,42	125,05	Not Analysed	Not Analysed
P056	47	Lung Malignancy	Lymph Node	Fragment	Red	32,08	19,96	Not Analysed	Not Analysed
P057	47	Lung Malignancy	Lymph Node	Fragment	Red	27,07	16,9	Not Analysed	Not Analysed
P058	48	Benign Disease	Lymph Node	Fragment	Blue	38,83	24,78	Not Analysed	Not Analysed
P059	48	Benign Disease	Lymph Node	Fragment	Orange	69,87	24,77	Not Analysed	Not Analysed
P060	50	Lung Malignancy	Pleura	Fragment	Orange	636,4	14,4	Not Analysed	Not Analysed

P061	50	Lung Malignancy	Pleura	Fragment	Orange	575,4	242,28	Not Analysed	Not Analysed
P062	50	Lung Malignancy	Pleura	Fragment	Orange	295,27	140,58	Not Analysed	Not Analysed

Table S3: *Multivariable logistic regression model evaluating factors independently associated with microplastic detection in thoracic tissue specimens. Age and smoking exposure were entered as continuous variables, and primary lung malignancy was entered as a binary categorical variable. Model fit statistics were as follows: omnibus test of model coefficients $\chi^2 = 13.18$ ($df = 3$, $p = 0.004$), Nagelkerke $R^2 = 0.318$, and Hosmer-Lemeshow goodness-of-fit test $p = 0.168$. Overall classification accuracy was 72.0%.*

Variable	β	SE	Wald χ^2	OR (95% CI)	p
Age (per year)	-0.072	0.030	5.719	0.93 (0.88-0.99)	0.017
Smoking exposure (per pack-year)	0.019	0.013	2.277	1.02 (0.99-1.04)	0.131
Primary lung malignancy	1.409	0.689	4.18	4.09 (1.06-15.79)	0.041
